# Developmental Transcriptome Profiling and WGCNA Identify Candidate Genes Associated with Sugar Accumulation and Fruit Enlargement in *Lycium ruthenicum*

**DOI:** 10.3390/genes17090986

**Published:** 2026-08-23

**Authors:** Yunzhang Xu, Xin Wu, Zhanwen Ma, Yu-E Ma, Zhenzhong Wu, Hengxia Yin, Shaoshan Zhang, Wenbing Li, Huachun Sheng

**Affiliations:** 1 State Key Laboratory of Plateau Ecology and Agriculture, Qinghai University, Xining 810016, China; 2023990008@qhu.edu.cn (Z.W.); hengxiayin@qhu.edu.cn (H.Y.); 2 College of Agriculture and Animal Husbandry, Qinghai University, Xining 810016, China; 15509723940@163.com; 3 Nanmenxia Forest Farm, Huzhu Tu Autonomous County, Haidong 810500, China; 13897636805@163.com; 4 Xi’an Agricultural Product Quality and Safety Inspection and Monitoring Center, Xi’an 710077, China; jieyougongzhu1314@163.com; 5 Sichuan Provincial Qiang-Yi Medicinal Resources Protection and Utilization Technology and Engineering Laboratory, Southwest Minzu University, Chengdu 610225, China; 80300101@swun.edu.cn (S.Z.); 26600005@swun.edu.cn (W.L.); 6 Tibetan Plateau Ethnic Medicinal Resources Protection and Utilization Key Laboratory of National Ethnic Affairs Commission of the People’s Republic of China, Southwest Minzu University, Chengdu 610225, China

**Keywords:** *L. ruthenicum*, fruit development, sugar accumulation, sugar transport, fruit enlargement, WGCNA

## Abstract

**Background:** *Lycium ruthenicum*, commonly known as Black goji berry, is a shrub species native to western China and is rich in bioactive compounds that contribute to its nutritional and health-promoting effects. However, the transcriptional dynamics associated with fruit development and quality formation in black goji berry remain incompletely understood. This study aimed to investigate transcriptional dynamics during fruit development and identify candidate genes associated with sugar accumulation and fruit enlargement. **Methods:** Fruits were sampled at five developmental stages (S1–S5), and RNA-seq was performed to investigate the alterations in gene expression. Differential expression analysis, temporal expression-pattern analysis, functional enrichment analysis, and weighted gene co-expression network analysis (WGCNA) were performed, followed by the integration of differential expression, temporal expression patterns, co-expression relationships, and functional annotation. **Results:** Fruit diameter, fresh weight, and total soluble sugar content increased markedly during the later developmental stages. Differential expression analysis revealed extensive transcriptional changes across successive developmental transitions, while temporal expression-pattern analysis identified distinct increasing and decreasing gene clusters. Functional enrichment analysis showed that the DEGs were mainly associated with carbohydrate metabolism, sugar transport, hormone-related processes, photosynthesis, and cell-wall modification. WGCNA identified 13 co-expression modules, among which MEyellow and MEbrown showed strong but opposite associations with developmental stage, total soluble sugar content, fruit diameter, and fruit fresh weight. The integrated analysis highlighted several candidate genes associated with sugar metabolism and transport during late fruit development. **Conclusions:** These findings provide a transcriptomic perspective on the coordinated changes in sugar accumulation and fruit enlargement during *L. ruthenicum* fruit development.

## 1. Introduction

*L. ruthenicum* has a long history of use in traditional medicine systems, particularly in Tibetan and Uighur medical practices. Traditional Tibetan medical texts such as *Jing Zhu Ben Cao* and *Si Bu Yi Dian* describe its application in treating a variety of health conditions, including cardiovascular diseases, menstrual disorders, and symptoms associated with menopause [1]. These historical records reflect the deep-rooted knowledge of the therapeutic potential of *L. ruthenicum* within indigenous medical traditions. Similarly, the *Pharmacography of Uighur Medicine* documents its use for urinary tract stones, fungal skin infections (tinea), abscesses (furuncles), and bleeding from the gums [1], indicating its broad utility in traditional healing practices.

Studies on the chemical composition of *L. ruthenicum* have identified a diverse range of bioactive compounds, such as flavonoids, anthocyanins, polysaccharides, phenolic acids, carotenoids, alkaloids, essential oils, and fatty acids [1,2]. Modern pharmacological studies have further reported various biological activities of *L. ruthenicum*, providing scientific support for some of its traditional uses. For example, *L. ruthenicum* exhibits strong antioxidant activity [3], which may contribute to its anti-aging effects [4]. Additionally, it has been reported to improve physical endurance and reduce fatigue [5], enhance immune function [6] and exert protection against radiation-induced damage [7]. These findings highlight the potential health-related value of *L. ruthenicum* and have contributed to increasing interest in its medicinal and functional properties.

One of the most notable features of *L. ruthenicum* fruit is its high anthocyanin content [8]. Most previous studies on *L. ruthenicum* have focused on its adaptation to abiotic stress and the regulatory networks associated with anthocyanin biosynthesis [9,10,11,12,13,14,15], while relatively little is known about the molecular changes associated with fruit development. Fruit quality in black goji berry is determined not only by anthocyanin accumulation but also by sugar synthesis and accumulation, which contribute substantially to fruit sweetness and consumer market preference. Zhao et al. (2015) systematically investigated changes in sugars and organic acids across five ripening stages of *Lycium barbarum* fruit and found that glucose and fructose contents increased markedly during ripening, whereas sucrose content gradually decreased [16]. They also reported total sugar and fructose contents were significant positively correlated with *L. barbarum* polysaccharide (LBP) content, suggesting a close association between sugar accumulation and fruit quality formation [16]. In addition, the sugar-to-acid ratio is an important indicator of fruit flavor quality and is closely associated with sensory characteristics and palatability [16,17].

In *L. ruthenicum*, sugar accumulation is an important determinant of fruit flavor and is also closely associated with anthocyanin glycoside biosynthesis, thereby contributing to overall fruit quality [18]. To better characterize the transcriptional changes associated with sugar accumulation and fruit enlargement, this study performed transcriptomic profiling across five fruit developmental stages. By integrating temporal expression pattern analysis, weighted gene co-expression network analysis (WGCNA) and fruit-related traits data, including total soluble sugar (TSS) content, fruit diameter, and fruit weight, we identified co-expression modules and candidate genes associated with sugar metabolism, sugar transport, and fruit enlargement. These analyses provide a transcriptional framework for understanding the coordinated changes associated with sugar accumulation and fruit development in *L. ruthenicum*.

## 2. Materials and Methods

### 2.1. Plant Material

**Figure 1:**
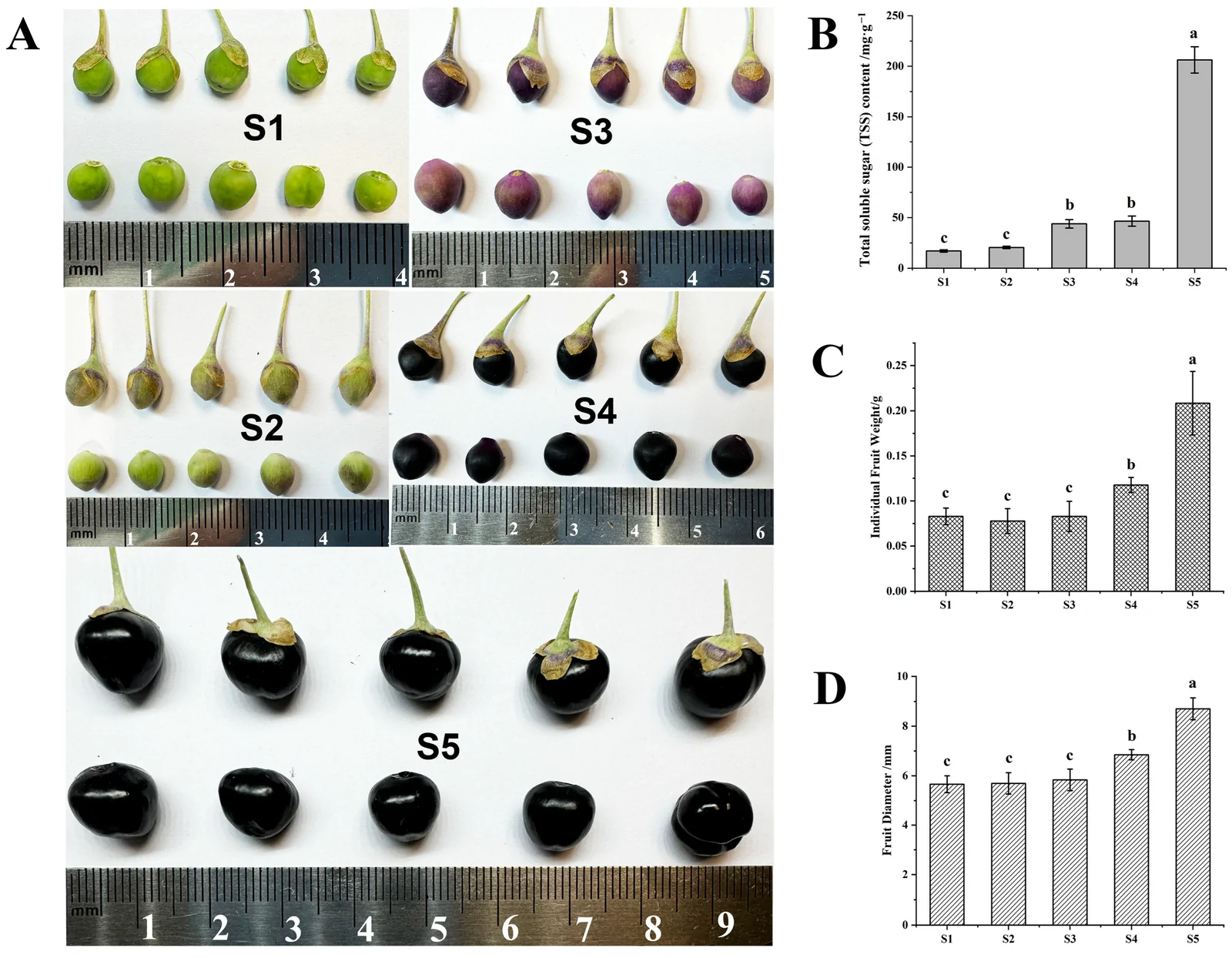
Phenotypic characteristics of *L. ruthenicum* fruits at different developmental stages. (**A**) Representative images of fruits at five developmental stages. S1, green fruit stage; S2, color transition stage I; S3, color transition stage II; S4 immature stage; S5, mature stage. (**B**–**D**) Dynamic changes in TSS (**B**), fresh fruit weight (**C**), and fruit diameter (**D**) during fruit development. Error bars represent standard deviations (*n* = 3 for TSS content and *n* > 10 for fresh fruit weight and fruit diameter). Different lowercase letters indicate significant differences among developmental stages (*p* < 0.05).

Fruits of *L. ruthenicum* at different developmental stages were collected in August 2024 from a cultivated field at Nuomuhong Farm, Dulan County, Haixi Mongolian and Tibetan Autonomous Prefecture, Qinghai Province (96°25′38.2″ E, 36°26′23.7″ N, altitude 2793 m). Based on continuous observations of fruit development by our research group and the developmental characteristics reported by Zhao et al. (2020) [19], five developmental stages were defined according to the combination of days after flowering and fruit morphological characteristics (Figure 1A). Because fruit development under natural conditions was not completely synchronous, the defined time intervals were used to accommodate variation in developmental rates. The five stages were defined as follows: green fruit stage (S1, 10–14 days after flowering), characterized by green fruits; color transition stage I (S2, 16–22 days after flowering), characterized by pigmentation covering approximately half of the fruit surface; color transition stage II (S3, 23–29 days after flowering), characterized by a uniform light-purple coloration over the fruit surface; immature stage (S4, 30–37 days after flowering), characterized by a deep-purple coloration; mature stage (S5, 38–45 days after flowering), characterized by fully developed and plump fruits. Three healthy and vigorously growing *L. ruthenicum* plants were selected, and sun-exposed branches were chosen for sampling. Within each developmental interval, fruits were selected according to the corresponding morphological criteria described above. The three plants were treated as three independent biological replicates. For each biological replicate, at least five fruits were collected from a single plant and pooled before RNA extraction. The number of fruits collected was adjusted according to fruit size at different developmental stages, with relatively more fruits sampled at the earlier developmental stages because of their smaller size. Fruits from different plants were not mixed. After removal of the persistent calyx, the samples were immediately placed in RNase-free cryotubes, flash-frozen in liquid nitrogen, and stored for subsequent experiments.

### 2.2. Quantification of TSS

TSS content was determined using the anthrone–sulfuric acid colorimetric method. Briefly, 1.0 g of *L. ruthenicum* fruit sample was homogenized with 5 mL of 80% (*v*/*v*) ethanol. An additional 15 mL of 80% ethanol was then added, and the mixture was extracted in a water bath at 80 °C for 30 min. After cooling to room temperature, the extract was centrifuged at 8500 rpm for 10 min. The supernatant was collected and quantitatively transferred to a 50 mL volumetric flask with 80% ethanol. The resulting extract was used for TSS determination. The colorimetric reaction was performed according to the anthrone-sulfuric acid method, and absorbance was measured at 620 nm using a spectrophotometer. A standard curve was constructed using serial dilutions of a glucose standard solution under the same experimental conditions, and TSS content was calculated accordingly.

### 2.3. Phenotypic Analysis

The weight of individual fruits was measured using an electronic analytical balance with a resolution of 0.01 g. Prior to weighing, surface moisture was meticulously removed with a clean, lint-free cloth to eliminate potential errors caused by residual water. For morphological assessment, high-resolution digital images were captured under controlled lighting conditions to ensure uniformity across samples. Fruit diameter was subsequently quantified using ImageJ (v1.51), an open-source platform widely adopted in scientific image analysis. More than 10 fruits per developmental stage were measured for both weight and diameter measurements.

### 2.4. Transcriptome Sequencing

Total RNA was extracted using TRIzol reagent according to the manufacturer’s instructions. RNA concentration and purity were assessed using a NanoDrop 2000 spectrophotometer (Thermo Fisher Scientific, Wilmington, DE, USA), and RNA integrity was evaluated using an Agilent 2100 Bioanalyzer (Agilent Technologies, Santa Clara, CA, USA). RNA-seq Libraries were constructed using the VAHTS Universal V5 RNA-seq Library Prep Kit (Vazyme Biotech Co., Ltd., Nanjing, China) according to the manufacturer’s instructions. The resulting libraries were sequenced on an Illumina NovaSeq 6000 platform. RNA sequencing and subsequent bioinformatics analyses were conducted by Shanghai OE Biotech Co., Ltd. (Shanghai, China).

### 2.5. Screening, Enrichment and Temporal Expression-Pattern Analysis of Differentially Ex Pressed Genes

Raw reads in FASTQ format were processed using fastp (v0.20.1) to remove low-quality reads and obtain clean reads for subsequent analyses. The clean reads were mapped to the *L. ruthenicum* H5 reference genome (Lycium_ruthenicum.H5.genome.fa), downloaded from the Figshare dataset “Wolfberry genomes and the evolution of *Lycium* (Solanaceae)” (dataset ID: 20416593; https://figshare.com/articles/dataset/Wolfberry_genomes_and_the_evolution_of_Lycium_Solanaceae_/20416593, accessed on 20 May 2025), using HISAT2 (v2.1.0). The corresponding genome annotation file (Lycium_ruthenicum.gff.filter3.rename.gff) was used for gene-level quantification. Gene expression levels were quantified as fragments per kilobase of transcript per million mapped reads (FPKM), and gene-level read counts were obtained using HTSeq-count (v0.11.2). Differential expression analysis was performed using DESeq2 (v1.22.2) [20] based on the gene-level read-count matrix. Genes with |log2FoldChange| ≥ 2 and an adjusted *p*-value (padj) ≤ 0.05 were considered differentially expressed genes (DEGs).

Pairwise differential expression analyses were performed among the five developmental stages. The four consecutive developmental-stage comparisons (S2 vs. S1, S3 vs. S2, S4 vs. S3, and S5 vs. S4) were used as the primary framework to characterize transcriptional changes between successive developmental stages. In addition, six non-adjacent comparisons (S3 vs. S1, S4 vs. S1, S5 vs. S1, S4 vs. S2, S5 vs. S2, and S5 vs. S3) were performed to assess cumulative transcriptional changes across broader developmental intervals. Gene Ontology (GO) and Kyoto Encyclopedia of Genes and Genomes (KEGG) enrichment analyses were performed based on the hypergeometric distribution to identify functional categories and pathways significantly enriched among the DEGs. The comparison-specific DEG sets from each of the four consecutive-stage comparisons were also subjected separately to GO and KEGG enrichment analyses.

Temporal expression-pattern analysis was performed using the Mfuzz package in R (v4.5.0). For each gene, the mean expression value of the three biological replicates at each developmental stage was calculated. The resulting five-stage expression matrix was standardized, followed by soft clustering to group genes exhibiting similar temporal expression patterns during fruit development.

### 2.6. WGCNA

WGCNA was performed using the WGCNA R package (v1.72-1) [21] independently of the differential expression analysis. The whole-gene FPKM expression matrix was used as the initial input. Genes with FPKM ≥ 1 in at least three of the 15 samples were retained, and the filtered expression values were transformed using log2(FPKM + 1). Genes with no expression variation across samples (median absolute deviation, MAD = 0) were removed. Finally, the 8000 genes with the highest MAD values were retained for network construction.

Candidate soft-thresholding powers ranging from 1 to 30 were evaluated based on scale-free topology fit and mean connectivity. The lowest power yielding a scale-free topology fit index (*R*^2^) ≥ 0.8 was selected, resulting in a soft-thresholding power of 12 for network construction. A signed hybrid co-expression network was constructed using biweight midcorrelation (bicor). Modules were identified with a minimum module size of 30, and modules with highly similar eigengene profiles were merged using a cut height of 0.25. Module eigengenes were subsequently calculated to summarize the overall expression profiles of individual modules.

Corresponding *p*-values were calculated using the corPvalueStudent function. For genes within the selected trait-associated modules, module membership (MM/kME) and gene significance (GS) were calculated using Pearson correlations to evaluate gene–module and gene–trait associations, respectively. A combined score (|MM| × |GS|) was calculated to rank genes within the corresponding modules. Because no predefined quantitative threshold was applied, this score was used only as an auxiliary ranking metric, and individual genes were not formally classified as hub genes. Module-associated candidate genes were further prioritized by integrating MM/kME and GS, temporal expression patterns, differential-expression evidence, and functional annotation.

### 2.7. qRT-PCR

To validate the RNA-seq expression profiles, ten key sugar-metabolism/transport genes were selected for qRT-PCR analysis, and the candidate genes prioritized for biological relevance were selected based on integrated results from DEG analysis and WGCNA. First-strand cDNA Synthesis SuperMix for qPCR (gDNA digester plus) kit (11141ES60; Yeasen Biotechnology, Shanghai, China). The qPCR was performed using Hieff ^®^ qPCR SYBR Green Master Mix (Low Rox Plus) kit (11201ES50; Yisheng Biotechnology, Shanghai, China) on an Applied Biosystems 7500 Real-Time PCR System. The amplification program consisted of an initial denaturation at 95 °C for 5 min, followed by 40 cycles of 95 °C for 10 s and 60 °C for 30 s, and a melting-curve analysis. *L. ruthenicum Actin* was used as the reference gene, and relative expression levels were calculated using the 2^−ΔΔCt^ method. Three biological replicates were analyzed for each developmental stage. Primer pairs were designed using the DNAMAN software (v6.0), and the primer sequences are provided in Table S1.

## 3. Results

### 3.1. Development Changes in Fruit Morphology and Soluble Sugar Content

As shown in Figure 1A, fruit morphology changed markedly across the five developmental stages. Fruits were predominantly green at S1 and S2, began to develop purple pigmentation at S3, and progressively deepened to dark purple or black at S4 and S5, accompanied by an evident increase in fruit size during the later developmental stages. Consistent with these developmental changes, TSS content exhibited a clear stage-dependent pattern (Figure 1B). It remained relatively low and showed no significant difference between S1 and S2, increased significantly at S3, stayed at a comparable level at S4, and then rose sharply to its highest level at S5. Fruit fresh weight and diameter showed similar but slightly delayed developmental patterns (Figure 1C,D). Both traits showed no significant differences among S1, S2, and S3, increased significantly at S4, and reached their highest values at S5. Collectively, these results suggested that the significant increase in TSS occurred earlier than the marked increases in fruit fresh weight and diameter, whereas all three traits reached their maximum levels at S5.

### 3.2. Transcriptome Sequencing and Differential Expression Analysis During Fruit Development

Fifteen fruit samples representing five distinct developmental stages of *L. ruthenicum* were subjected to Illumina sequencing. After filtering and quality control, a total of 133.23 Gb of clean data was obtained, with 7.76–11.58 Gb of clean data and more than 52.14 million clean reads generated per sample (Table S2). The Q30 percentage ranged from 97.25% to 97.71%, and the average GC content was 44.31%. The mapping rate to the reference genome ranged from 73.49% to 80.55%, with uniquely mapped reads accounting for 63.35% to 71.53%.

**Figure 2:**
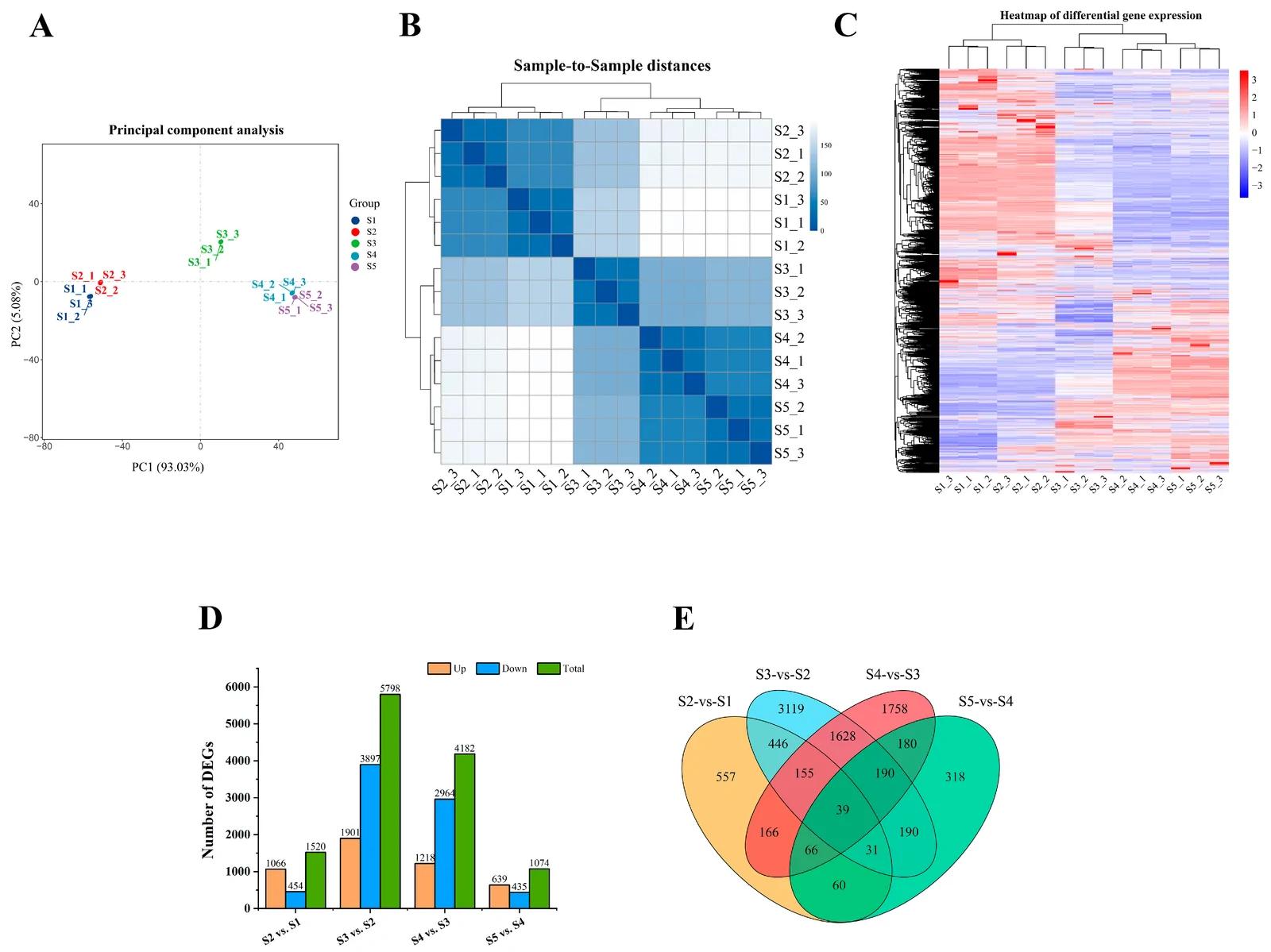
Transcriptome analysis during *L. ruthenicum* fruit development. (**A**) Principal component analysis (PCA) of samples from five developmental stages. (**B**) Heatmap of sample-to-sample distances. (**C**) Hierarchical clustering heatmap of DEGs. (**D**) Numbers of upregulated, downregulated, and total DEGs in four consecutive stage comparisons. (**E**) Venn diagram showing unique and shared DEGs among the four comparison groups. Different colors indicate S2 vs. S1, S3 vs. S2, S4 vs. S3, and S5 vs. S4.

Principal component analysis (PCA) showed that PC1 and PC2 explained 93.03% and 5.08% of the total variance, respectively, accounting for 98.11% of the transcriptomic variation (Figure 2A). Biological replicates from the same developmental stage clustered closely. S1 and S2 were positioned relatively close to each other, as were S4 and S5, whereas S3 occupied an intermediate position between the early and late developmental stages. Sample-distance analysis showed a similar clustering pattern, with biological replicates from the same stage clustering closely together (Figure 2B). Hierarchical clustering of differentially expressed genes (DEGs) further grouped replicates from the same developmental stage and revealed distinct stage-dependent expression patterns (Figure 2C). Overall, these analyses demonstrated good reproducibility among biological replicates and clear transcriptional differences across developmental stages.

Differential expression analysis was first performed for four consecutive developmental-stage comparisons (Figure 2D). a total of 1520 DEGs were identified in S2 vs. S1, including 1066 upregulated and 454 downregulated genes. S3 vs. S2 showed the largest number of DEGs, with 5798 genes, including 1901 upregulated and 3897 downregulated genes. S4 vs. S3 contained 4182 DEGs, comprising 1218 upregulated and 2964 downregulated genes, whereas S5 vs. S4 contained 1074 DEGs, including 639 upregulated and 435 downregulated genes. A Venn diagram analysis identified 557, 3119, 1758, and 318 unique DEGs in S2 vs. S1, S3 vs. S2, S4 vs. S3, and S5 vs. S4, respectively, with 39 DEGs shared among all four comparisons (Figure 2E). Further examination of these 39 shared DEGs revealed predominantly stage-dependent rather than monotonic expression patterns (Figure S1, Table S3). Only *Lru01G008670.1* (*UGT74G1*) showed a consistently increasing expression pattern, whereas a germin-like protein gene (*Lru01G000810.1*) consistently decreasing pattern; the remaining 37 genes exhibited non-monotonic trajectories. These shared DEGs included *SWEET11* and *C/VIF1*, together with genes annotated as encoding alpha-glucosidase, polygalacturonase, and XTH proteins, as well as hormone-related genes such as *GH3.1*, *GASA14*, and *ERF017*.

To complement the consecutive-stage comparisons, six non-adjacent comparisons were also performed (Table S4). The number of DEGs ranged from 4643 in S5 vs. S3 to 9380 in S5 vs. S1. Comparisons spanning broader developmental intervals generally yielded larger numbers of DEGs, particularly S4 vs. S1, S5 vs. S1, S4 vs. S2, and S5 vs. S2, in which 8893–9380 DEGs were identified. Notably, downregulated genes outnumbered upregulated genes in all six non-adjacent comparisons, revealing extensive transcriptional changes across fruit development.

**Figure 3:**
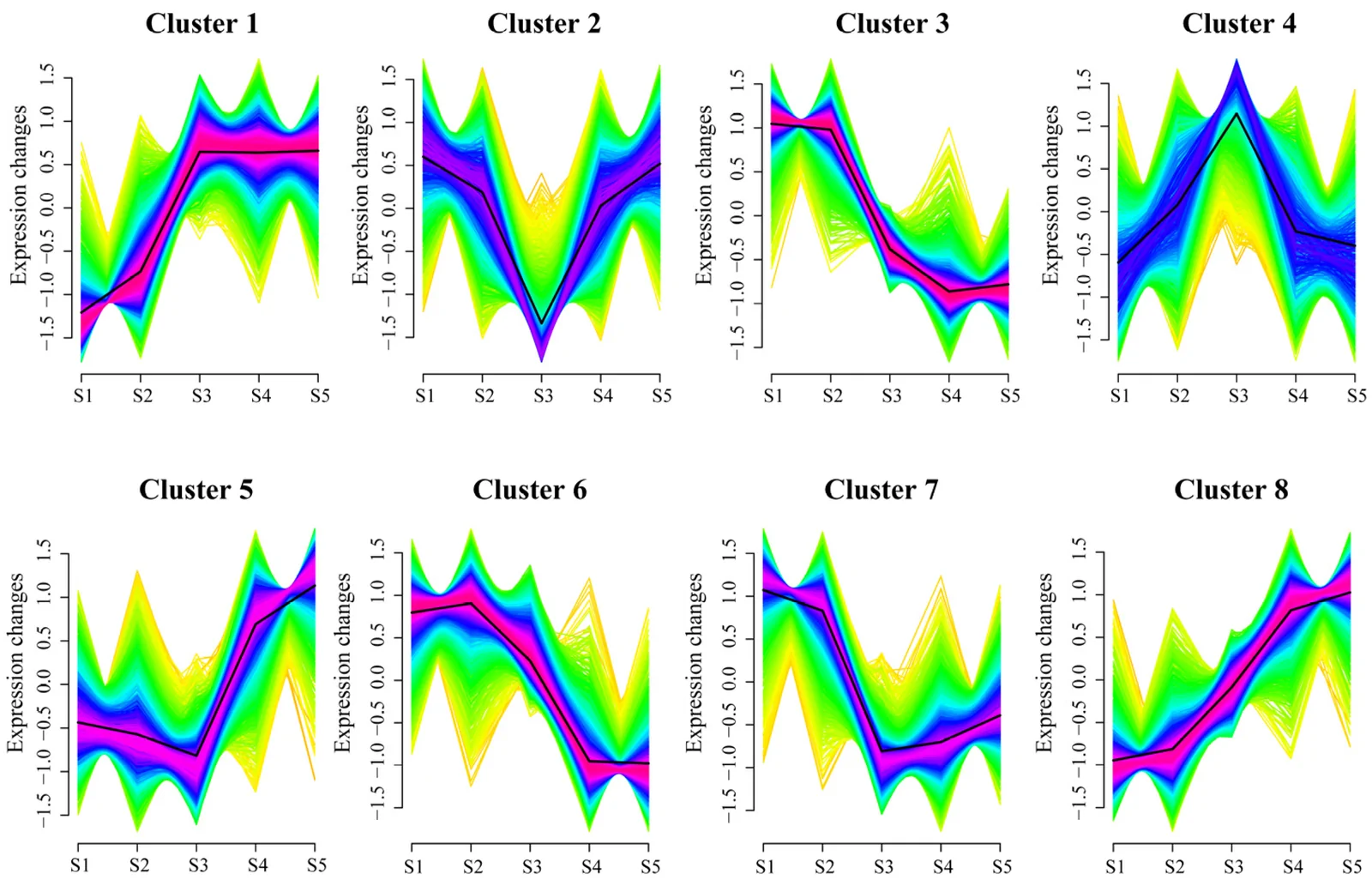
Temporal expression -pattern clustering of DEGs during *L. ruthenicum* fruit development. Colored lines represent individual gene expression profiles, and black lines represent the mean expression patterns of each cluster.

Mfuzz clustering separated the analyzed genes into eight temporal expression clusters (Figure 3). Clusters 1, 5, and 8 showed progressively increasing expression patterns and contained 1531, 958, and 1486 genes, respectively, for a total of 3975 genes (Table S5). In contrast, Clusters 3 and 6 showed decreasing expression patterns and contained 2701 and 1803 genes, respectively, totaling 4504 genes (Table S6). Notably, the increasing clusters included multiple genes associated with sugar transport and carbohydrate metabolism, such as *SWEET16*, *SWEET5*, *SWEET1*, *STP13*, *SPS*, *SUS2*, *PFK2/3/5*, and *HXK2*, together with several XTH, expansin, and aquaporin genes. Other members of these functional families, including *SWEET11/12/14*, *SUC4*, *STP8/14*, and several XTH and expansin genes, were present in the decreasing clusters, revealing heterogeneous temporal expression patterns among functionally related genes during fruit development.

### 3.3. Functional Enrichment of DEGs Across Consecutive Developmental Transitions

**Figure 4:**
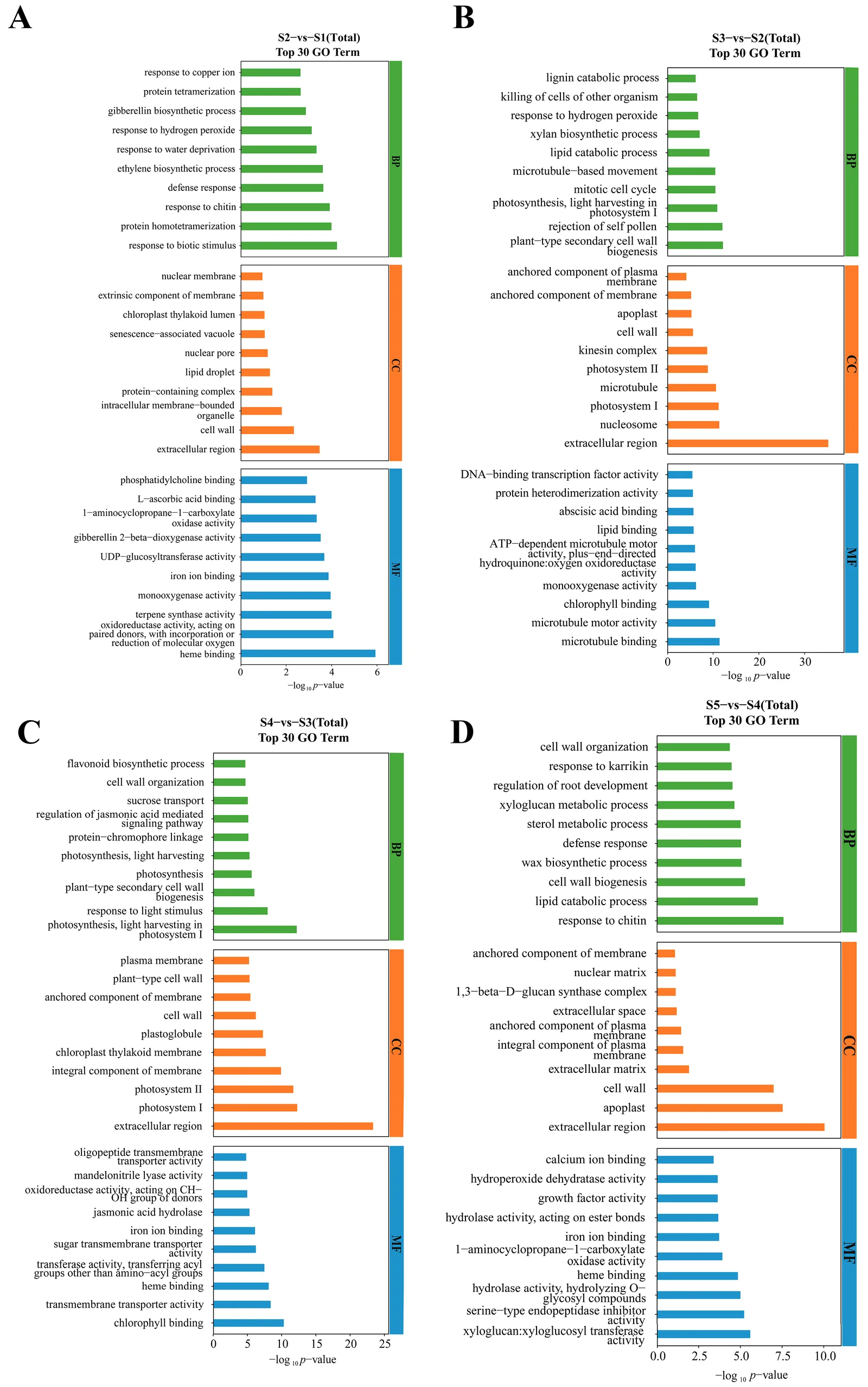
Gene Ontology (GO) enrichment analysis of differentially expressed genes (DEGs) across four consecutive developmental-stage comparisons: (**A**) S2 vs. S1; (**B**) S3 vs. S2; (**C**) S4 vs. S3; and (**D**) S5 vs. S4.

**Figure 5:**
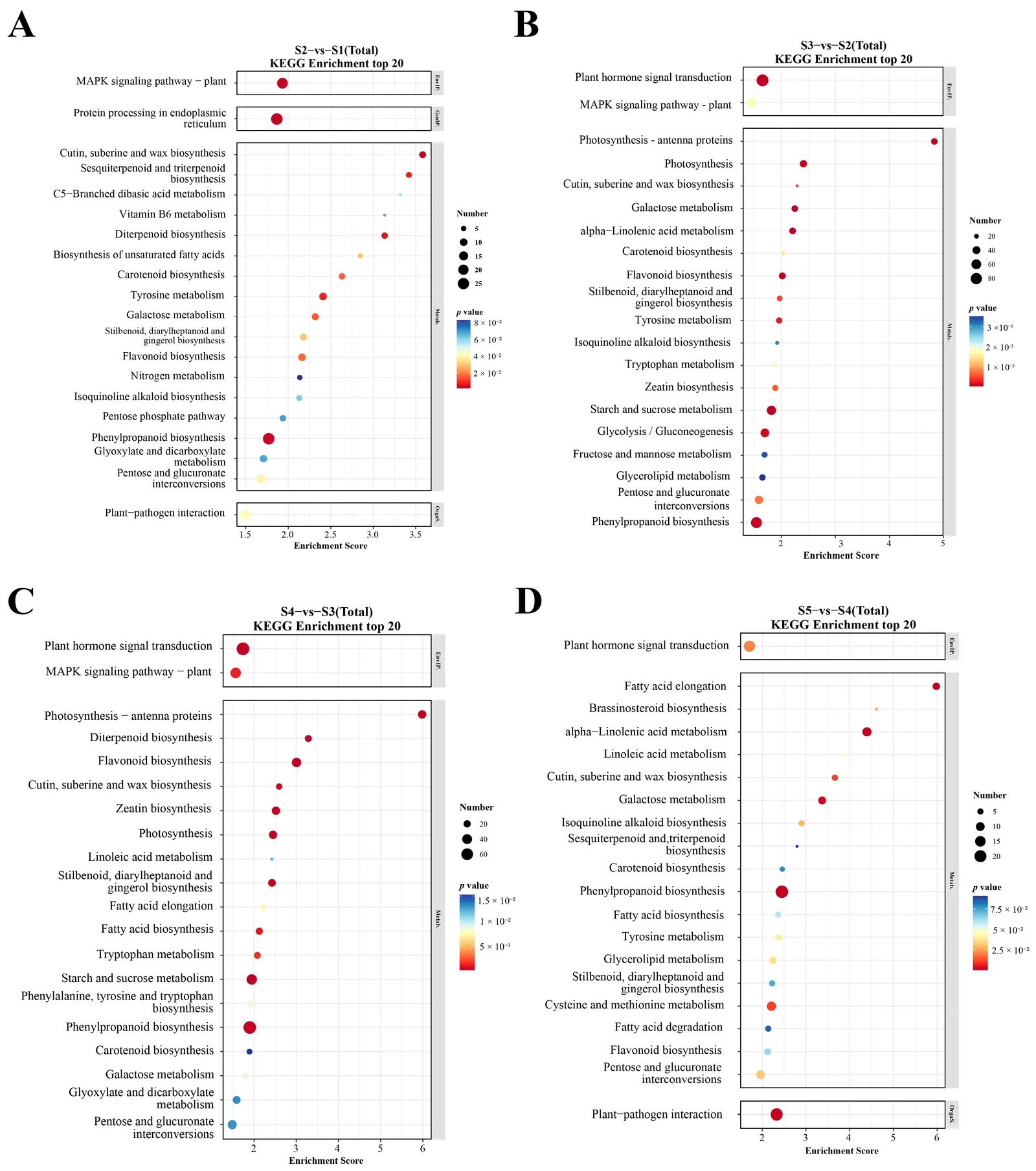
Comparative KEGG metabolic pathway enrichment analysis of DEGs. (**A**) S2 vs. S1. (**B**) S3 vs. S2. (**C**). S4 vs. S3 (**D**) S5 vs. S4.

GO and KEGG enrichment analyses were performed for DEGs identified in each of the four consecutive developmental-stage comparisons (S2 vs. S1, S3 vs. S2, S4 vs. S3, and S5 vs. S4) to characterize functional changes during fruit development (Figure 4 and Figure 5). In the S2 vs. S1 comparison, prominent GO biological process (BP) terms were related to gibberellin and ethylene biosynthesis, whereas the extracellular region was prominent in the cellular component (CC) category and heme binding in the molecular function (MF) category (Figure 4A). KEGG analysis showed enrichment in pathways including plant MAPK signaling, phenylpropanoid biosynthesis, and flavonoid biosynthesis (Figure 5A).

In the S3 vs. S2 comparison, enriched GO terms were mainly associated with plant hormone-related processes and cell-wall synthesis and remodeling (Figure 4B). KEGG analysis also identified enrichment in the plant MAPK signaling pathway and plant hormone signal transduction pathways (Figure 5B), indicating substantial transcriptional changes in genes related to these processes during this developmental transition.

In the S4 vs. S3 comparison, BP enrichment was characterized by photosynthesis-related processes, including light harvesting in photosystem and response to light stimulus, together with terms associated with cell-wall modification and sugar transport (Figure 4C). Enriched CC terms included the extracellular region, photosystem I, and photosystem II, whereas prominent MF terms included chlorophyll binding and transmembrane transporter activity. KEGG enrichment showed corresponding changes in pathways associated with this developmental transition (Figure 5C).

In the S5 vs. S4 comparison, GO enrichment was characterized by a greater representation of cell-wall-related processes and functions (Figure 4D). Prominent MF terms included xyloglucosyl transferase activity, hydrolase activity acting on O-glycosyl compounds, and hydrolase activity acting on ester bonds. KEGG analysis further identified plant hormone-related and secondary-metabolism pathways, with brassinosteroid biosynthesis among the represented pathways (Figure 5D).

To further characterize transcriptional changes specific to individual developmental transitions, GO and KEGG enrichment analyses were conducted separately for the comparison-specific DEGs from the four consecutive comparisons (Figures S2 and S3). In S2 vs. S1, enriched functions were mainly associated with hormone responses, cell-cycle-related processes, signal transduction, and primary metabolism. In S3 vs. S2, comparison-specific DEGs were associated with cellular glucose homeostasis and hexokinase activity and were enriched in carbohydrate-related pathways, including starch and sucrose metabolism, fructose and mannose metabolism, galactose metabolism, and glycolysis/gluconeogenesis. The S4 vs. S3 comparison showed enrichment of terms related to primary cell-wall biogenesis, transmembrane transport, water-channel activity, and carotenoid biosynthesis, together with pathways associated with starch and sucrose metabolism, phenylpropanoid biosynthesis, flavonoid biosynthesis, and anthocyanin biosynthesis. In S5 vs. S4, cell-wall-related terms, including cell wall organization, cell wall biogenesis, xyloglucan metabolism, and xyloglucosyl transferase activity, were particularly prominent. Overall, the comparison-specific DEG sets exhibited distinct functional enrichment patterns across successive developmental transitions, highlighting marked transcriptional changes in carbohydrate metabolism and transport, cell-wall-related processes, and other developmental pathways during stages characterized by increasing sugar accumulation and fruit enlargement.

### 3.4. Development-Associated Co-Expression Modules Identified by WGCNA

**Figure 6:**
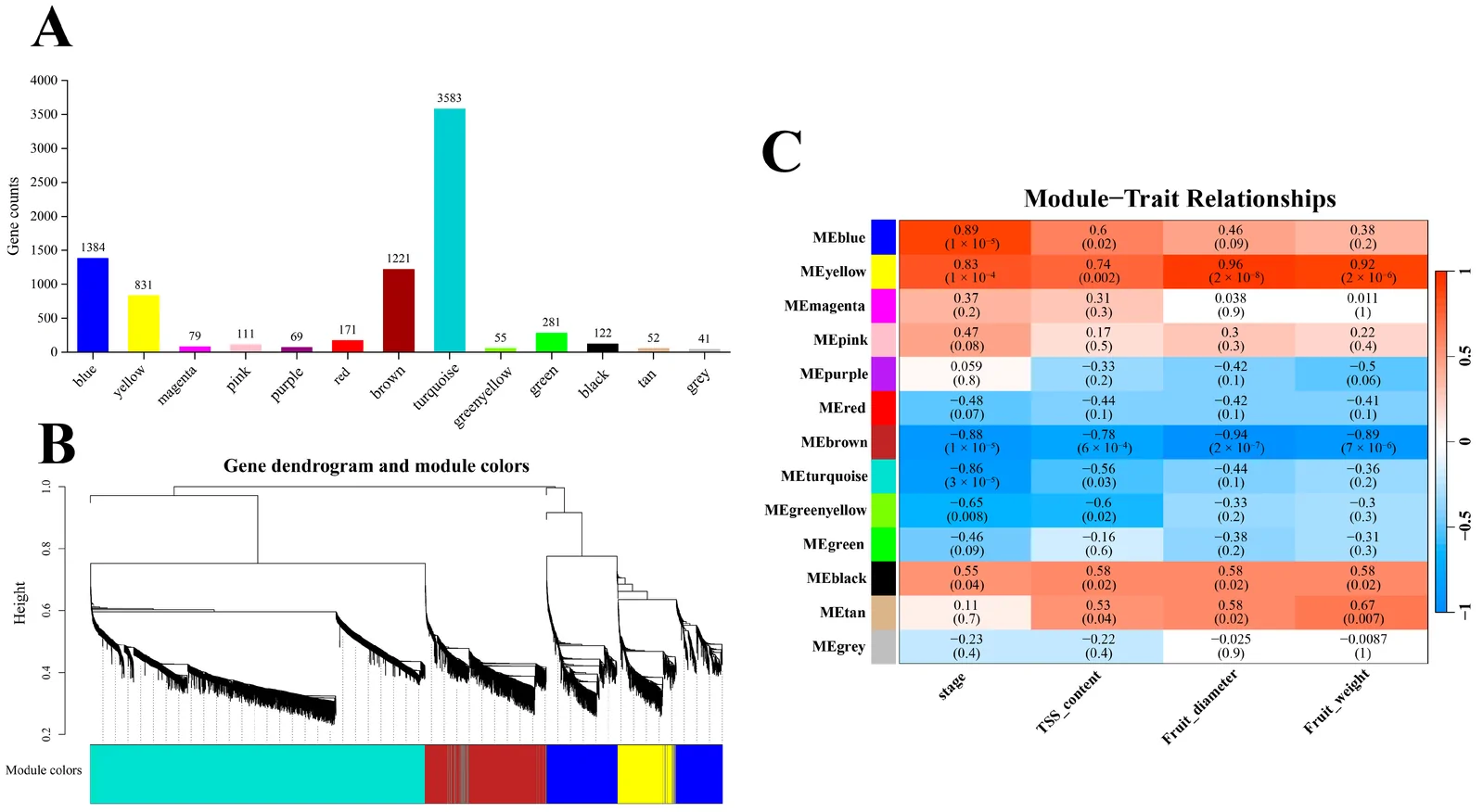
WGCNA of gene co-expression modules and module–trait relationships during *L. ruthenicum* fruit development. (**A**) Number of genes assigned to each co-expression module, with different colors representing distinct modules. (**B**) Hierarchical clustering dendrogram of genes used for WGCNA, with different colors representing distinct co-expression modules. (**C**) Module–trait relationships between module eigengenes and developmental stage, TSS content, fruit diameter, and fruit weight. Pearson correlation coefficients and corresponding *p* values are shown in each cell, with red and blue indicating positive and negative correlations, respectively.

WGCNA identified 13 co-expression modules across the 15 fruit transcriptome samples (Figure 6A,B; Table S7). Module-trait correlation analysis showed that several modules were associated with developmental stages, TSS content, fruit diameter, and fruit weight (Figure 6C). Among these modules, MEyellow showed positive correlations with developmental stage (r = 0.83, *p* = 1 × 10^−4^), TSS content (r = 0.74, *p* = 0.002), fruit diameter (r = 0.96, *p* = 2 × 10^−8^), and fruit weight (r = 0.92, *p* = 2 × 10^−6^). In contrast, MEbrown was negatively correlated with developmental stage (r = −0.88, *p* = 1 × 10^−5^), TSS content (r = −0.78, *p* = 6 × 10^−4^), fruit diameter (r = −0.94, *p* = 2 × 10^−7^), and fruit weight (r = −0.89, *p* = 7 × 10^−6^). Based on the strength and consistency of these associations, MEyellow, containing 831 genes, and MEbrown, containing 1221 genes, were selected for subsequent candidate gene prioritization. Because developmental stage, TSS content, fruit diameter, and fruit weight changed concurrently during fruit development, these modules were interpreted as development-associated co-expression modules whose expression profiles covaried with the measured fruit traits rather than as trait-specific regulatory modules. Candidate genes within these modules were subsequently prioritized by integrating module membership (kME), gene significance (GS), temporal expression patterns, differential-expression evidence, and functional annotation.

### 3.5. Developmental Expression Patterns of Sugar Metabolism and Transport Related Candidate Genes During Fruit Development

**Figure 7:**
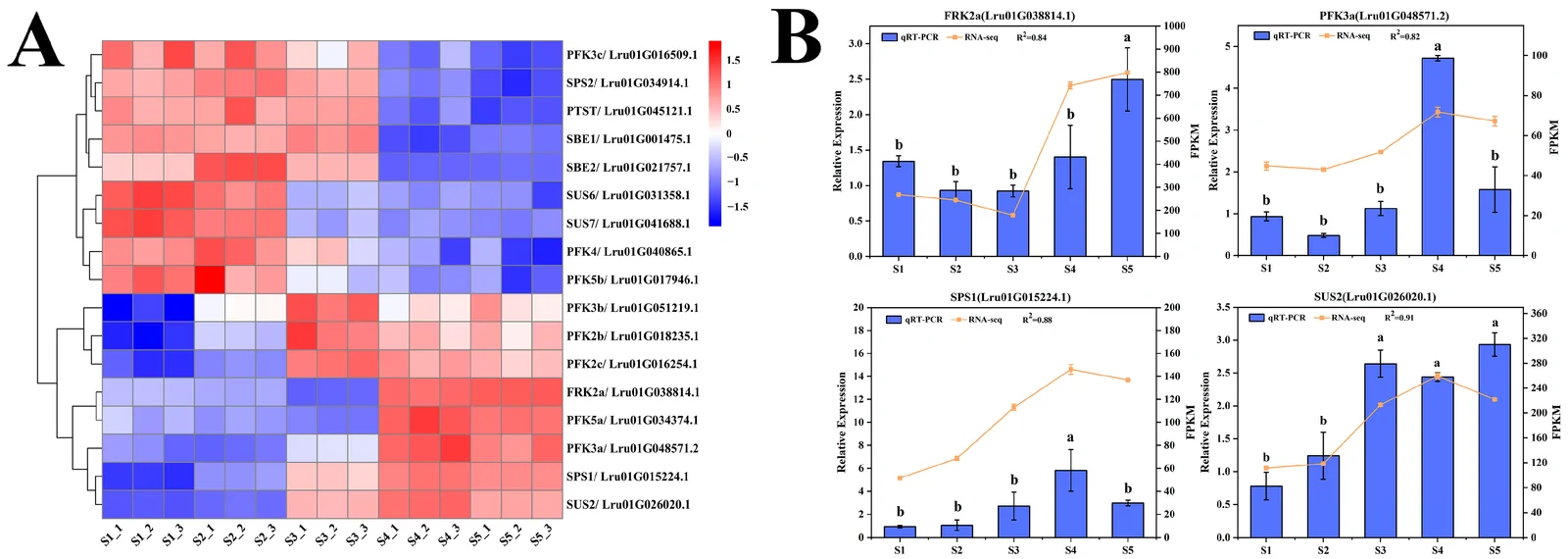
Developmental expression patterns of sugar metabolism related candidate genes in *L. ruthenicum* fruits. (**A**) Heatmap showing the expression profiles of sugar metabolism related candidate genes across five developmental stages. (**B**) qRT-PCR validation of selected candidate genes. Error bars represent standard deviations (*n* = 3). *R*^2^ indicates the coefficient of determination between the RNA-seq and qRT-PCR expression profiles. Different lowercase letters indicate significant differences among developmental stages (*p* < 0.05).

Based on functional annotation and developmental expression profiles, several candidate genes associated with sugar metabolism exhibited distinct temporal expression patterns (Figure 7). *LrSUS6* (Sucrose Synthase 6), *LrSUS7* (Sucrose Synthase 7), *LrSPS2* (Sucrose Phosphate Synthase 2), *LrPFK4* (Phosphofructokinase 4), and *LrSBE1* (Starch Branching Enzyme 1) showed relatively high expression during the early developmental stages (S1–S3). In contrast, *LrSUS2* (sucrose synthase 2), *LrSPS1* (sucrose-phosphate synthase 1), *LrPFK2* (phosphofructokinase 2), *LrPFK3* (phosphofructokinase 3), and *LrPFK5* (phosphofructokinase 5) showed higher expression during the later developmental stages (S4–S5). Expression profiles revealed distinct stage-dependent transcriptional patterns among carbohydrate metabolism-related genes during fruit development. Notably, the increased expression of several of these genes at S4–S5 coincided with increases in TSS content, fruit diameter, and fruit fresh weight during the later developmental stages.

**Figure 8:**
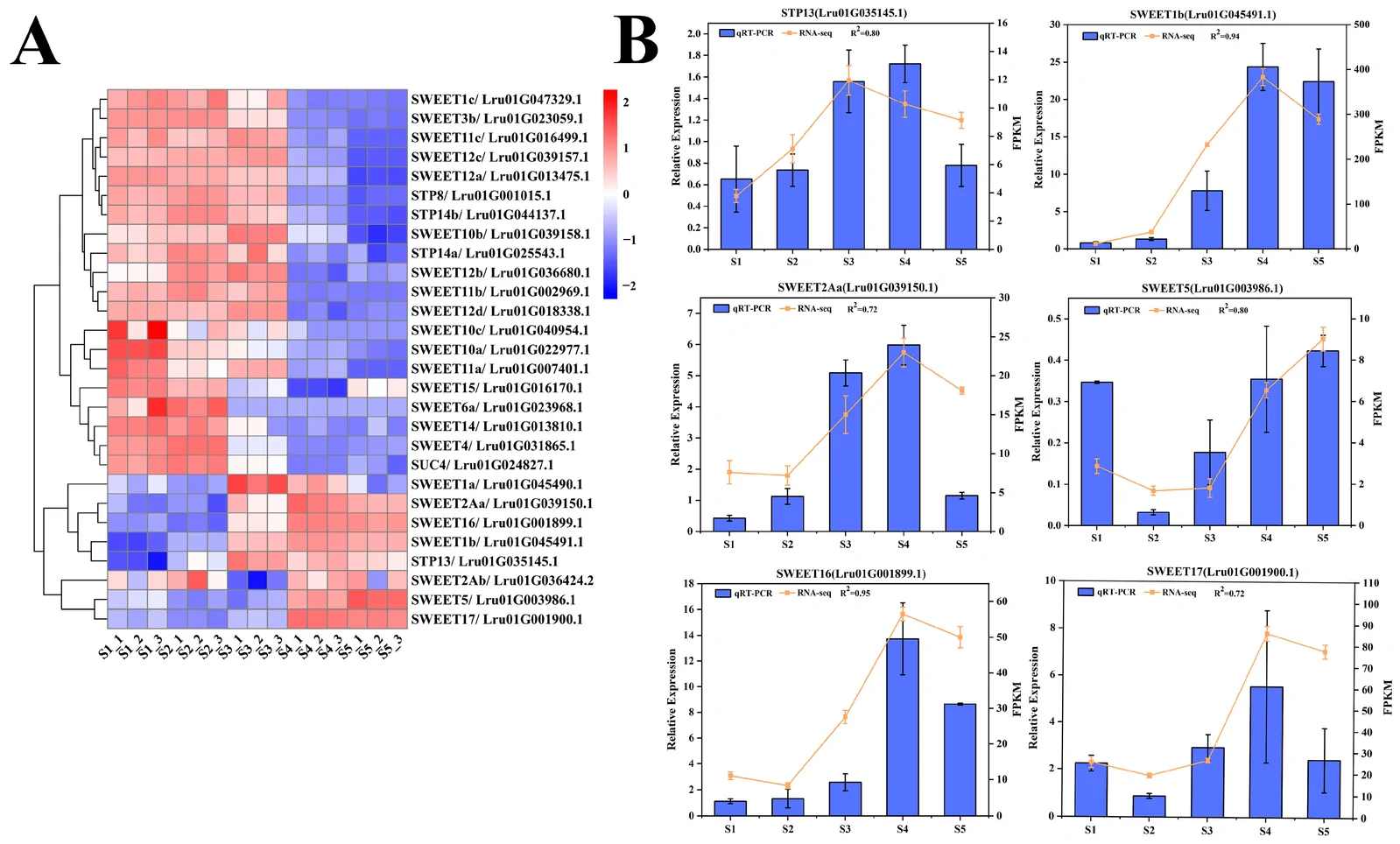
Developmental expression patterns of sugar transporter related candidate genes in *L. ruthenicum* fruits. (**A**) Heatmap of showing the expression profiles of sugar transporter related candidate genes across five developmental stages. (**B**) qRT-PCR validation of selected candidate genes. Error bars represent standard deviations (*n* = 3). *R*^2^ indicates the coefficient of determination between the RNA-seq and qRT-PCR expression profiles. Different lowercase letters indicate significant differences among developmental stages (*p* < 0.05).

Sugar transport in plants is mediated by several transporter families, among which STP, SWEET, and SUC/SUT proteins are involved in sugar uptake, transport, and distribution [22]. Candidate genes encoding these sugar transporters also displayed distinct developmental expression patterns in *L. ruthenicum* fruits (Figure 8). Most of the examined sugar transporter genes exhibited relatively high expression during S1–S3, whereas *LrSTP13*, *LrSWEET5*, *LrSWEET16*, and *LrSWEET17* are predominantly expressed during the stages S4 and S5. These contrasting expression profiles indicate temporal differentiation among sugar transporter genes during fruit development, with several candidates showing increased expression during the later stages characterized by higher TSS content and pronounced fruit enlargement. Overall, the RNA-seq and qRT-PCR expression profiles showed good concordance across the ten validated genes, with coefficients of determination (*R*^2^) ranging from 0.72 to 0.95 (Figure 7 and Figure 8).

## 4. Discussion

### 4.1. Sugar Accumulation and Fruit Quality Formation in Black Wolfberry

Soluble sugar is important component of fruit quality in black wolfberry, contributing to sensory characteristic, color formation, and functional nutritional properties during fruit ripening. Glucose and fructose constitute major soluble sugars in mature black wolfberry fruits, whereas sugar composition changes substantially during ripening, jointly affecting the sugar–acid balance and fruit taste and flavor [23]. In *L. ruthenicum,* sugar metabolism is also closely associated with anthocyanin biosynthesis. Hexoses not only serve as important carbon sources, but also provide UDP-sugar substrates for anthocyanin glycosylation mediated by *LrUFGT* and other glycosyltransferases [24]. The concurrent accumulation of hexoses and anthocyanins during ripening suggests a close relationship between sugar availability and anthocyanin accumulation, which contributes to the characteristic purple-black pigmentation and antioxidant properties of mature fruits [18].

Environmental conditions such as salinity and water availability can influence sugar metabolism and carbohydrate partitioning, resulting in variation in fruit quality traits among different habitats [25]. Recent spatio-temporal metabolomic studies have further revealed tissue-specific distribution patterns of soluble sugars between the fruit pericarp and seeds, indicating heterogeneous sugar allocation within developing fruits [26]. Collectively, these findings suggest that sugar accumulation is closely associated with both primary and secondary metabolism during fruit development, and a better understanding of the relationship between sugar metabolism and quality formation may provide a theoretical basis for quality-oriented cultivation of black wolfberry.

Our study revealed pronounced changes in the expression of genes associated with sugar metabolism, sugar transport, and photosynthesis-related processes during the S3-to-S4 transition (Figure 4C). During the early developmental stages (S1–S2), photosynthesis provides an important source of carbohydrates for plant growth and development. Sucrose, as a major photosynthetic product in many plant species, is the principal form of carbon transported from photosynthetically active source tissues, such as mature leaves, to sink tissues, including development fruit, primarily through the phloem [27]. Consistent with this source–sink relationship, several carbohydrate metabolism-related genes, including *LrSUS6*, *LrSUS7*, *LrSPS2*, *LrPFK4*, and *LrSBE1*, together with many sugar transporter genes, exhibited relatively high expression during S1–S3 (Figure 7 and Figure 8). These expression patterns suggest substantial transcriptional activity associated with carbohydrate metabolism and sugar transport during the early stages of fruit development.

Following phloem unloading into sink tissues, sucrose can enter different metabolic pathways depending on the plant species, tissue type, and developmental stage. Invertases hydrolyze sucrose into glucose and fructose [27,28], whereas sucrose synthase catalyzes the reversible conversion of sucrose and UDP into UDP-glucose and fructose. Previous studies have reported that sucrose is relatively abundant in black wolfberry fruits before color transition, whereas glucose and fructose increase markedly after fruit coloration [29]. Because the present study measured total soluble sugar rather than individual sugar species, these compositional changes cannot be directly inferred from our data. Nevertheless, several carbohydrate metabolism-related genes, including *LrSUS2*, *LrSPS1*, *LrPFK2*, *LrPFK3*, and *LrPFK5*, together with sugar transport genes such as *LrSTP13*, *LrSWEET5*, *LrSWEET16*, and *LrSWEET17*, showed relatively high expression during S4 and S5 (Figure 7 and Figure 8). SUS, SPS, and PFK participate in different aspects of carbohydrate metabolism, including reversible sucrose metabolism, sucrose biosynthesis, and glycolysis, respectively, while sugar transporters contribute to sugar uptake and distribution [22,28]. Their developmental expression patterns therefore suggest transcriptional reorganization of carbohydrate metabolism and sugar transport during late fruit development rather than direct evidence of sucrose-to-hexose conversion. In particular, the possible intracellular sugar-transport functions of *LrSWEET16* and *LrSWEET17* remain hypotheses based on sequence homology and expression patterns. Overall, the DEGs associated with carbohydrate metabolism and sugar transport identified in this study provide candidate genes and transcriptional evidence for further investigating sugar accumulation and fruit-quality formation in *L. ruthenicum*.

### 4.2. Potential Associations of Soluble Sugars with Osmotic Regulation, Sugar Signaling, and Fruit Enlargement

Cell expansion is a major component of fruit enlargement and is closely associated with sugar transport and metabolism as well as cell wall loosening and remodeling [29,30,31]. During S4 and S5, the diameter and fresh weight of *L. ruthenicum* fruits increased markedly, accompanied by transcriptional changes in genes associated with sugar metabolism and transport, brassinosteroid (BR) biosynthesis, and cell wall modification (Figure 4, Figure 5, Figure 7 and Figure 8). Soluble sugars can contribute to cellular osmotic adjustment, while turgor pressure provides an important physical basis for plant cell expansion [32]. In mature fruit cells, substantial amounts of soluble sugars can be compartmentalized within vacuoles, which are pivotal structures for cellular osmotic homeostasis [28]. Therefore, vacuole sugar accumulation has been proposed to contribute to osmotic adjustment and cell expansion during fruit growth [28]. In *Arabidopsis*, AtSWEET16 and AtSWEET17 are localized to the tonoplast and participate in vacuolar sugar transport [33,34]. In the present study, *LrSWEET16* and *LrSWEET17* showed relatively high expression during S4 and S5. Based on their sequence homology and developmental expression patterns, these genes may be candidates associated with intracellular sugar partitioning during late fruit development. However, their subcellular localization, transport properties, and possible relationship with fruit enlargement remain to be experimentally validated.

In addition to their metabolic and osmotic functions, sugars can also act as signaling molecules. Changes in sugar availability can be perceived through several conserved sugar-sensing and signaling components, including HXK1 (hexokinase 1), RGS1 (regulator of G-protein signaling 1), TPS1 (trehalose-6-phosphate synthase 1), TOR (target of rapamycin), and SnRK1 (SNF1-related protein kinase 1) [35,36,37]. These components link cellular sugar status with downstream metabolic and developmental responses. For instance, AtHXK1 has been reported to influence F-actin dynamics, cytoskeleton-associated polysomes, and vesicular trafficking processes related to cell expansion in *Arabidopsis* [38]. These findings provide a possible framework for considering how developmental changes in sugar metabolism may be associated with cell-expansion-related processes. Nevertheless, sugar-signaling activity was not directly measured in the present study, and its involvement in *L. ruthenicum* fruit enlargement therefore remains hypothetical.

Brassinosteroids (BRs), as key plant hormones, play an essential role in regulating cell expansion [39], and interactions between BR and sugar signaling have been well documented in previous studies [40,41,42]. In *Arabidopsis*, sugar and BR signaling can influence hypocotyl cell elongation through transcriptional changes in cell wall related genes, including *EXPA17*, *EXPLA2*, and *XTH6* [43]. Our previous study also demonstrated that sucrose affected the BIN2-BZR1 signaling module through the TOR–S6K2 pathway and was accompanied by changes in cellulose synthesis, hypocotyl cell elongation in *Arabidopsis* [44]. In the present study, the concurrent increases in TSS content and fruit size during late development, together with transcriptional changes in genes associated with sugar metabolism, BR-related pathways, and cell-wall remodeling, suggest a possible coordination among these processes in *L. ruthenicum*. However, the present data do not establish a causal sugar–BR–cell-wall regulatory pathway. Instead, this relationship should be regarded as a working hypothesis that will require physiological, biochemical, and genetic validation.

## 5. Conclusions

This study systematically characterized transcriptional changes across five developmental stages of *L. ruthenicum*. Integrated analyses identified development-associated co-expression modules and candidate genes related to sugar metabolism, sugar transport, and cell-wall remodeling, whose expression patterns were associated with increasing soluble sugar content and fruit enlargement during late development. These findings provide a transcriptional framework for understanding sugar accumulation and fruit development in *L. ruthenicum* and identify candidate genes for further investigation. Further genetic, biochemical, and physiological validation will be required to establish their specific functions and regulatory relationships.

## Data Availability

The RNA-seq data generated in this study have been deposited in the CNSA at https://db.cngb.org/cnsa/ (accessed on 20 May 2025) under accession number CAR029153. Other data supporting the findings of this study are available within the article and its Supplementary Materials. Additional information may be obtained from the corresponding author upon reasonable request.

## Supplementary Materials

The following supporting information can be downloaded at: https://www.mdpi.com/article/10.3390/genes17090986/s1, Figure S1: Temporal expression patterns of the 39 DEGs shared across all four consecutive developmental-stage comparisons; Figure S2: GO enrichment analyses of comparison-specific DEGs during consecutive developmental transitions; Figure S3: KEGG enrichment analyses of comparison-specific DEGs during consecutive developmental transitions; Table S1 Primer sequences used in this study; Table S2 Transcriptome Data Summary; Table S3 Functional annotation and expression profiles of the 39 DEGs shared across all four consecutive developmental-stage comparisons; Table S4: Differentially expressed genes identified in non-adjacent developmental-stage comparisons of *Lycium ruthenicum* fruit; Table S5 Information on genes in continuously increasing expression clusters identified by trend analysis; Table S6 Information on genes in continuously decreasing expression clusters identified by trend analysis; Table S7 Gene assignments to the 13 WGCNA modules; Table S8: WGCNA-associated candidate genes during fruit development.
